# Diets of white‐headed langurs (*Trachypithecus leucocephalus*) inhabiting limestone forests: The effects of habitat fragmentation and implication for conservation

**DOI:** 10.1002/ece3.11716

**Published:** 2024-07-09

**Authors:** Ying Lai, Yanqiong Chen, Kechu Zhang, Zhonghao Huang

**Affiliations:** ^1^ Key Laboratory of Ecology of Rare and Endangered Species and Environmental Protection (Guangxi Normal University), Ministry of Education Guilin China; ^2^ Guangxi Key Laboratory of Rare and Endangered Animal Ecology Guangxi Normal University Guilin China; ^3^ The Chongzuo White‐Headed Langur Field Observation and Research Station of Guangxi Chongzuo China; ^4^ Key Laboratory of Mountain Biodiversity Conservation, Education Department of Guangxi Zhuang Autonomous Region Yulin Normal University Yulin China

**Keywords:** diet, habitat fragmentation, karst forest, *Trachypithecus leucocephalus*, white‐headed langurs

## Abstract

Information about wildlife diets is crucial for comprehending how species adapt to varying environments in fragmented habitats and for developing effective conservation strategies. White‐headed langurs (*Trachypithecus leucocephalus*) are exclusively found in fragmented limestone forests in southwestern China. To investigate the effects of habitat fragmentation on langurs' diets, we collected published dietary data and relevant environmental factors spanning from 1996 to 2021 at two regions with different degrees of fragmentation (Banli > Bapen), from 10 studies (three of Banli and seven of Bapen). The results demonstrated that the diets of white‐headed langurs were significantly influenced by environmental factors, including habitat fragmentation, annual rainfall, and mean annual temperature. Food item diversity index was significantly and positively affected by the fragmentation index, the higher fragmentation the langurs suffered, the more diverse food items they consumed. Besides, fruit consumption was negatively influenced by annual rainfall and the consumption of other items was influenced by mean annual temperature. Notably, although there are no significant differences in the feeding proportions of food items or food item diversity indices were observed between the Banli and Bapen groups, the Banli groups extensively consumed ground‐supported kudzu (*Pueraria montana* var. *lobata*), a plant rarely recorded in the dietary preferences of the Bapen groups, implying that the large plants likely lacking in the fragmented limestone forests. Our findings provide evidence of the major impact of habitat fragmentation on the dietary composition of white‐headed langurs, highlighting the need of considering the possibility that the habitats of the white‐headed langurs have all undergone extreme fragmentation, inferring the conservation efforts should prioritize protecting native vegetation and reducing human disturbance.

## Graphical abstract

Our findings underscore the impact of habitat fragmentation on the white‐headed langurs' diets, emphasizing the importance of maintaining native vegetation and reducing human disturbance in developing effective conservation strategies for langurs.

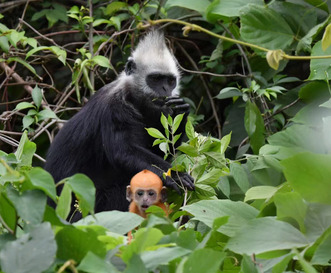

## 1 INTRODUCTION

Habitat fragmentation restricts wildlife survival and development by reducing the size of wildlife habitat patches and increasing their isolation. This phenomenon is particularly evident in most forest‐dwelling mammals, such as some primates, as the forest vegetation is the one directly affected and they are usually the shelter and food on which the animals depend (Arroyo‐Rodríguez & Dias, 2010; Crooks et al., 2017; Estrada et al., 2017). The population size and distribution of primates are directly affected by the availability of food resources within their habitat. Consequently, alterations in foraging strategies serve as crucial indicators of primate adaptability (Freeland, 1979; Gould et al., 1999; Machovsky‐Capuska et al., 2016). Changes in habitat quality have a profound impact on the distribution of food resources, compelling primates to adjust their foraging behavior (Petersen et al., 2023). In response to decreased habitat quality, primates may exhibit increased dietary flexibility, which more commonly includes increased feeding on an expanded range of food species/food items (*Alouatta palliata*: Cristóbal‐Azkarate & Arroyo‐Rodríguez, 2007; *Chlorocebus djamdjamensis*: Mekonnen et al., 2018), or choosing to predominantly feed on relatively lower‐quality/fallback foods (*Eulemur collaris*: Donati et al., 2011; *Ateles hybridus*: de Luna et al., 2017; *Alouatta palliata*: Dunn et al., 2012; *Theropithecus gelada obscurus*: Tesfaye et al., 2021). In addition, some primates living in fragments near agricultural zones tend to consume large amounts of crops or cultivated plants (*Cercopithecus mitis boutourlinii*: Tesfaye et al., 2013; *Pan troglodyte*: McLennan et al., 2020; *Macaca sylvanus*: Neves et al., 2024).

Climatic conditions also play a crucial role in influencing the foraging strategies of primates, particularly on a large geographic scale (Tsuji et al., 2013, 2015). Spatial variations in climatic factors affect the distribution of food resources, resulting in variations in the dietary composition of primates (Tsuji et al., 2013). Rainfall and temperature, in particular, are key indicators of the primary productivity of a region (Rosenzweig, 1968). Studies on Asian macaques have revealed that annual rainfall and mean annual temperature significantly influence their feeding behavior, leading to regional differences in dietary composition. For instance, macaques rely on more fruits in tropical regions but more leaves in temperate regions (Rosenzweig, 1968; Tsuji et al., 2013). When comparing the dietary composition of narrowly distributed primates with similar climatic conditions, it is essential to consider the effects of changes in habitat quality. For example, Hainan gibbons (*Nomascus hainanus*), which are exclusively distributed in the Bawangling Forest Area of China, have lower proportions and diversity of large edible plants in secondary forests than populations in mountain forests (Zhang et al., 2022). The population living in the secondary forest is indirectly compelled to choose alternative food items because secondary forests have fewer large trees due to deforestation, and the large trees contribute to the supply of fruits (Zhang et al., 2022).

It has been shown that some primates do not consistently choose preferred or high‐quality food but actively select other food items to maintain a nutritional balance (DiGiorgio et al., 2023; Lambert & Rothman, 2015). However, this behavior also needs to be based on the availability of food resources in the primate's current habitat (Knott, 2005). When the environment is favorable and resourceful, these primates may have easier access to food with the appropriate nutritional value (Knott, 2005). Otherwise, they could be driven to consume large quantities of higher productivity but relatively lower‐quality food (de Luna et al., 2017; Knott, 2005). In tropical regions, compared to other food items, fruits are relatively high‐quality food for their ability to supply energy quickly and their easily digestible structure (Sengupta & Radhakrishna, 2016). Besides, they are susceptible to changes in rainfall and temperature owing to their growth dynamics (Dunham et al., 2018; Joly et al., 2023). Tropical primates could adjust their feeding proportions of fruits based on alterations in fruit resources under varying rainfall and temperatures (Martinez et al., 2022). For instance, three cercopithecines in a rainforest in Gabon prioritize fleshy fruits during the rainy season and decrease the proportions of leaf and seed feeding (Brugiere et al., 2002). Notably, tropical rainforests can provide a sufficient fruit‐feeding base for primates even in habitats with reduced quality, as the black‐and‐white ruffed lemurs (*Varecia variegata*) in Madagascar can maintain high fruit consumption in fragmented forests by searching for a wider range of fruit species (Petersen et al., 2023). These findings underscore the importance of considering regional specificity, i.e., whether the region can offer a stable foundation for plant growth, when exploring the determinants of primate dietary composition.

Karst represents a distinctive geological landform, differing from typical earthen hills because of its limestone composition, characterized by thin soils, limited surface water, and low vegetation biomass (Jiang et al., 2014). Karst landscapes often experience severe soil erosion, as rainfall causes surface soil and water to flow through crevices, exposing the underlying bedrock (Bai et al., 2013). The limestone hills predominantly feature cliffs, and their steep slopes contribute kinetic energy to soil erosion, intensifying the process further (Bailey et al., 1998). The absence of vegetation on exposed rocks eliminates their temperature buffering function, leading to significant surface temperature variations (Guha & Govil, 2021; Kranjc, 2012). Additionally, the isolation between limestone hills exacerbates habitat fragmentation (Wang et al., 2004). These distinctive features of karst landscapes may prompt inhabiting primates to develop more specialized adaptive strategies, particularly in their search for food and water (Huang et al., 2015, 2017). Owing to low soil formation rates and high permeability, karst areas have become vulnerable to damage from human activities (Jiang et al., 2014; Wang et al., 2004), such as resource extraction, arable land cultivation, urban expansion, and road construction. These activities amplify the negative impacts of fragmentation on the karst ecosystem, directly or indirectly influencing the adaptation strategies of inhabiting primates (Huang et al., 2017; Jiang et al., 2014).

The white‐headed langur (*Trachypithecus leucocephalus*), a member of the Colobinae family, holds a critically endangered status according to the International Union for Conservation of Nature (Bleisch & Long, 2020). This species is exclusively found in an area less than 200 km^2^ in Chongzuo, Guangxi, southwestern China, comprising three fragments: the Nongshan area of the Nonggang Nature Reserve, the Chongzuo area, and the Fusui area of the Chongzuo White‐headed Langur National Nature Reserve (Guangxi Forestry Bureau, 2011; Huang et al., 2008). Influenced by the subtropical monsoon climate, the langurs' habitat is characterized by simultaneous rain and heat (Huang, 2002). Edible vegetation types in limestone forests are more abundant during the rainy season than during the dry season (Lu et al., 2016; Tang et al., 2016). Moreover, their habitat faces additional fragmentation because of proximity to human‐cultivated sugarcane fields (Huang et al., 2008). Frequent human activities and habitat fragmentation pose significant threats to the survival and development of these langurs (Huang et al., 2017; Tang et al., 2023). Previous studies have revealed that langurs in fragmented habitats or during dry seasons tend to increase their intakes of fiber‐rich foods and rely on a wider range of food categories compared to those in less fragmented forests and/or during rainy seasons (Li & Elizabeth, 2006; Zhou et al., 2013). Foraging information from white‐headed langurs can contribute to a more direct understanding of their adaptive strategies in fragmented habitats.

In this study, we aimed to gain a more comprehensive understanding of the dietary ecology of the white‐headed langur, a species narrowly distributed in fragmented limestone forests, and to further develop conservation plans. We collected all dietary data (from 1996 to 2021) of these langurs in the Banli and Bapen patches of the Chongzuo White‐headed Langur National Nature Reserve on the basis of langur groups. To assess the adaptability of the langurs to karst environments, we examined the correlation based on the entire species between their feeding proportions of food items and environmental variables, including the fragmentation index, annual rainfall, and mean annual temperature. Additionally, we determined the impact of habitat fragmentation on the foraging strategies of the langurs in different regions by investigating regional differences in feeding proportions of food items between the Banli and Bapen patches. Furthermore, we explored differences in specific plant species that consumed by the langurs between these two regions to help improve conservation strategies for their feeding vegetation.

## 2 MATERIALS AND METHODS

### 2.1 Study sites and subjects

**FIGURE 1:**
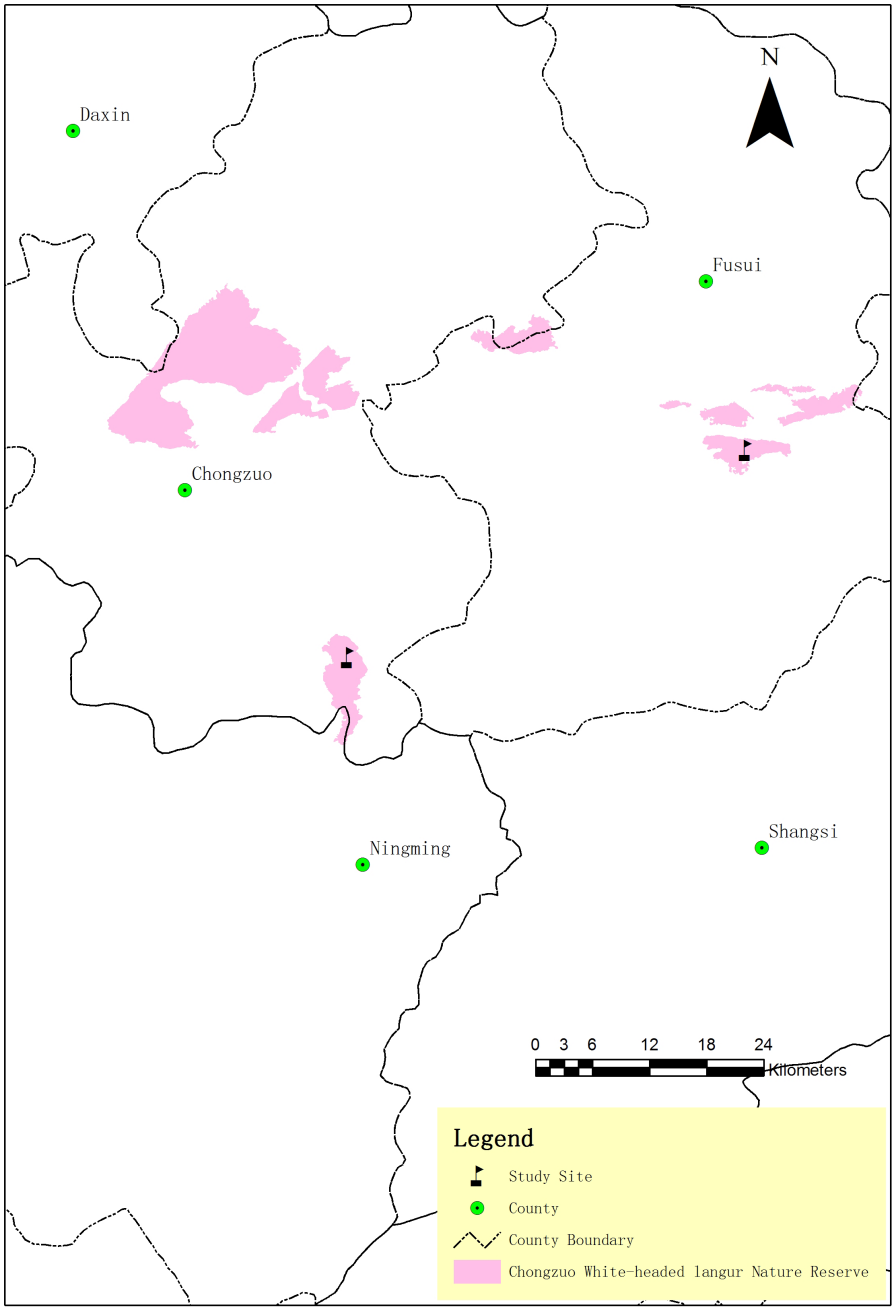
Locations of the study sites in the Chongzuo White‐headed Langur National Nature Reserve. The locations of the two study sites are marked by black flags: Banli is in the Chongzuo County, whereas Bapen is in the Fusui County (cited and modified from Huang et al., 2017).

White‐headed langurs are primarily found in the Guangxi Chongzuo White‐headed Langur National Nature Reserve (107°16′53″ to 107°59′46″ E, 22°10′43″ to 22°36′55″ N) in southwest Guangxi, China (Figure 1). This reserve includes four patches: Banli and Tuozhu of the Chongzuo County, Bapen of the Fusui Country, and Dalin at the boundary between the Chongzuo County and the Fusui Country (Figure 1) (Tang et al., 2023). In particular, the Banli and Bapen patches of this reserve have the densest distribution of langurs, accounting for 94% of the total distribution (Tang et al., 2023). All study sites mentioned in these collected articles were concentrated in the Banli and Bapen patches (Figure 1). This reserve features a typical karst forest and experiences seasonal variations, with an altitude range of 400–600 m above sea level (Guangxi Forestry Department, 1993). The size of Banli and Bapen patches are 20.84 km^2^and 29.35 km^2^, respectively (Huang et al., 2008). The straight‐line distance between the edges of these two areas is approximately 42 km. The limestone hills of Banli and Bapen are isolated, and habitat fragmentation in these two areas is severe because of human activities such as reclamation, grazing, and road construction (Huang et al., 2008). The fragmentation indices (FIs) for Banli and Bapen are 0.9733 and 0.9665 respectively, larger value indicates higher fragmentation (Feng, 2005; Huang et al., 2008). These indices are calculated using the following formula: $\mathrm{FI}=1-\sum_{i=1}^{n}\frac{1}{D_{i}}\cdot \left(\frac{A_{i}}{A}\right)$, where *n* denotes the number of patches, *A*_*i*_ refers to the area of the *i*‐th patch, *A* refers to the total area of the study site, and *D*_*i*_ denotes the patch shape index (Feng, 2005). Besides, thin soil covers the slopes of the rocky hills, and rainfall seeps down through the crevices, resulting in low vegetation biomass. Of these, the vegetation diversity of Banli was lower than that of Bapen (vegetation diversity index: 3.44 vs. 5.49) (Huang et al., 2017; Huang & Li, 2005). Bapen langur groups were found to have more available arboreal resources and feed on fewer lianas compared to Banli langur groups (Huang et al., 2017).

### 2.2 Literature search and selection

All articles on white‐headed langurs were searched on the Web of Science (https://www.webofscience.com/wos/) and the China National Knowledge Infrastructure (CNKI, https://www.cnki.net/) using the keywords ‘white‐headed langur,’ ‘*T. leucocephalus*,’ and ‘white‐headed black langur’ in English and Chinese. To avoid analyzing duplicated data and overlooking important information, we excluded several publications that used the same data from the same study period and location. Because we focused on geographic variation, we ensured that the observation period for all studies spanned 8 months. This method allowed us to capture the changing patterns of food items in both dry and rainy seasons, and is already in a stable period that no new species consumed by langurs are being observed (Zhang, 2018). A total of 23 studies reported on the consumption of food items by wild white‐headed langurs, 10 of which were selected for the final analysis (three of Banli and seven of Bapen).

### 2.3 Data collection and selection

The articles that were searched were reviewed individually to gather data on the use of food items and climatic data in the aforementioned habitats, including any information presented in figures and tables.

We collected as much environmental data as possible from existing articles to evaluate the impact of the environment on dietary composition. Only annual rainfall and mean annual temperature are the climate information commonly found in every article. We collected these information and used the descriptions provided by the authors in their original article or obtained from the European Centre for Medium‐Range Weather Forecasts (https://www.ecmwf.int/). When the observation period is less than 12 months, we collected data from all months and treated them as annual data. Additionally, specialized investigation reports on habitat quality were found because of the fragmentation of limestone habitats for white‐headed langurs. We used the habitat FI and human disturbance index from a study by Huang et al. (2008). Although the FIs were tested in 2005, they can already meet the basic needs of data analysis. This is because the management department can only control the destruction of hills in the protected area but cannot regulate the development activities of farmers on flat land at the foothills (Guangxi Forestry Bureau, 2011). Therefore, the living area pattern of the langurs, which mainly operates on steep hills, has not changed significantly across these years. In other words, the degree of fragmentation in each of the two study sites has not changed much over time. To evaluate the covariance of each environmental factor using the variance inflation factor, objects with values exceeding 10 units were excluded from the analysis. Finally, three factors, including the annual rainfall (2.34), the mean annual temperature (1.57), and habitat FI (2.10), were included in further analysis (Huang et al., 2008).

We found that the categorizations of food items in some studies were general. To preserve as much analyzable data as possible, we categorized the food items in this study, including leaves (including young and mature leaves), fruits (including immature and mature fruits), flowers, and other items (such as bark, seeds, petioles, buds, stems, roots, and unidentifiable parts).

To assess the dietary composition of white‐headed langurs, the food item diversity index (FIDI) was calculated using the Shannon–Wiener index (*H*′) as follows: $H^{\prime }=-\sum P_{i}\cdot \log P_{i}$, where *P*_*i*_ denotes the proportions of food items (Begon et al., 2006; Tsuji et al., 2015). A larger *H*′ value indicates a higher degree of diversity.

Instead of averaging the feeding proportions of food items across all langur groups in the original article and conducting analyses based on individual studies, which may have excluded information on the langurs and led to incomplete analyses, food item data that were directly collected from each langur group in the study were used whenever feasible. Huang et al. (2000), Tang (2004), Zhou et al. (2013), and Huang (2021) only provided the overall mean value for all langur groups. Information from the articles for analysis is provided in Table 1. Multiple lines from the same study in the consumption of food items (%) indicate each langur group dietary data collected from that study. For this study, 10 studies were used (three of Banli and seven of Bapen), and dietary data was collected from 22 langur groups (seven of Banli and 15 of Bapen).

**TABLE 1:** References relevant to the dietary composition of white‐headed langurs found in the literature search.

Study site		References	Study period	Climate data		FDI	FS	NG	Consumption of food items (%)				FIDI
Name	FI			MT (°C)	AR (mm)				Leaves	Fruits	Flowers	Others	
Banli	0.9733	Li et al. (2016)	2011.08–2012.07	22.60	1240.30	4.11	134	2	72.36	22.80	3.10	1.90	0.328
									80.78	11.40	5.80	2.20	0.291
		Zhang (2018)	2016.09–2017.08	22.50	4382.90	4.96	181	4	86.50	6.60	1.10	5.80	0.226
									86.60	4.80	2.40	6.20	0.231
									86.50	6.90	1.60	5.10	0.229
									83.10	8.10	3.80	4.90	0.273
		Huang (2021)	2019.07–2020.01, 05‐08	26.16	1312.63	2.60	51	2	73.20	10.30	6.40	10.10	0.378
Bapen	0.9665	Huang et al. (2000)	1995.04–1996.12	22.10	1022.00	—	—	6	75.26	22.46	2.28	0.00	0.276
		Li (2000)	1997.09–1998.09	25.00	1168.00	3.20	50	6	88.90	6.60	0.00	4.40	0.183
									98.70	1.20	0.00	0.00	0.029
									93.20	3.00	2.80	1.10	0.139
									68.90	22.50	4.50	4.10	0.375
									89.20	2.00	6.90	2.00	0.192
									88.00	8.10	1.40	2.50	0.203
		Tang (2004)	2002.07–2003.06	22.10	1022.00	—	—	3	82.22	15.89	1.67	0.12	0.230
		Huang (2009)	2007.07–2008.08	23.20	1204.25	3.61	107	3	85.43	8.17	3.88	2.53	0.306
									83.37	12.42	3.45	0.68	0.408
									91.06	5.95	1.31	1.69	0.300
		Zhou et al. (2013)	2002.07–2003.06	22.10	1022.00	2.70	109	3	91.60	4.20	0.30	3.90	0.155
		Liao et al. (2021)	2012.01–12	25.30	663.25	—	—	1	77.00	15.10	5.10	1.60	0.242
		Lu et al. (2021)	2013.01–12	25.30	977.00	4.02	104	1	68.30	15.70	6.60	8.50	0.244
			2016.01–12	22.90	1022.00	3.48	70	1	77.20	17.20	2.20	3.00	0.165

Abbreviations: AR, Annual rainfall; FDI, Food diversity index (based on plant species); FI, Fragmentation index; FIDI, Food item diversity index (based on food items); FS, Number of food species; MT, Mean annual temperature; NG, Number of langur groups.

### 2.4 Data analysis

To ensure the normality and linearity of the selected data, variables presented as percentages were transformed using the arcsine square root method, whereas those not presented as percentages were transformed using the log(*X* + 1) method (Li, Ma, Zhou, & Huang, 2020). All analyses were performed using R 4.2.1.

To investigated the correlation between the feeding proportions of the food items consumed by white‐headed langurs and relevant environmental factors, we used the generalized linear model (GLM) to test the effect of environmental factors on food item composition when not being grouped by regions (based on the entire species) (Li, Ma, Zhou, & Huang, 2020). In this model, the feeding proportions of food items (%) and the FIDI were separately set as response variables. The annual rainfall, mean annual temperature, and FI were set as explanatory variables. The value of *p* < .05 was considered to indicate that the explanatory variables significantly affected the response variables (Li, Ma, Zhou, & Huang, 2020).

Although the distance between the two study sites is relatively close, we considered that their FIs differ and further investigated whether there are significant regional differences in the consumption of food items. Additionally, we used the generalized linear mixed model (GLMM) to compare regional differences (Li, Ma, Zhou, Li, & Huang, 2020). In the model, the study sites were set as a fixed factor, the feeding proportions of food items (%) as response variables, and the observation periods for each langur group as random factors (Li, Ma, Zhou, Li, & Huang, 2020). ANOVA was used to compare the differences between the two models with and without fixed effects to determine the effect of fixed factors on the response variables (Li, Ma, Zhou, Li, & Huang, 2020). The value of *p* < .05 was considered  indicator of a statistically significant difference between the two study sites.

To conduct our inquiry on a finer scale, we further selected eight studies containing the feeding proportions of specific food species consumed by langurs (Table 1). Additionally, we used the Mann–Whitney *U*‐test to compare regional differences in the number of plant species consumed by the langurs and the food diversity index (FDI). To assess whether the specific plant species consumed by the langurs exhibited regional differences, we calculated the average feeding proportions of the top 10 plants in each study. We selected the top 10 plants because they were all identifiable by the observer and because of the limitations imposed by how some studies presented their results.

## 3 RESULTS

The GLM regression analysis revealed that FIDI was significantly and positively affected by FI (*β* = 30.42 and *p* = .035). Fruit consumption was negatively influenced by annual rainfall (*β* = −0.36 and *p* = .022). The consumption of other items was influenced by mean annual temperature (*β* = 1.67, *p* = .030; Table 2). These results indicate that the FI, annual rainfall, and mean annual temperature significantly influence the feeding proportions of food items used by white‐headed langurs.

**TABLE 2:** Effect of environmental factors on the diets of white‐headed langurs in limestone forests based on the GLM.

Response variable	Explanatory variable	Estimate (*β*)	SE	*t*‐Value	*p*‐Value
Leaves	Intercept	31.11	14.80	2.10	.049*
	MT	0.25	1.14	0.22	.826
	AR	0.31	0.16	2.01	.060
	FI	−106.35	50.94	−2.09	.051
Fruits	Intercept	−17.90	13.57	−1.32	.204
	MT	−1.59	1.04	−1.53	.144
	AR	−0.36	0.14	−2.50	.022*
	FI	73.26	46.73	1.57	.134
Flowers	Intercept	−18.92	9.60	−1.97	.064
	MT	0.67	0.74	0.91	.378
	AR	−0.14	0.10	−1.40	.178
	FI	63.22	33.04	1.91	.072
Others	Intercept	−16.43	9.25	−1.78	.093
	MT	1.67	0.71	2.35	.030*
	AR	0.12	0.10	1.19	.251
	FI	47.28	31.84	1.49	.155
FIDI	Intercept	−8.75	3.87	−2.26	.037
	MT	0.08	0.30	0.27	.787
	AR	−0.07	0.04	−1.72	.104
	FI	30.42	13.32	2.28	.035*

Abbreviations: AR, annual rainfall; FI, fragmentation index; FIDI, Food item diversity index (based on food items); MT, mean annual temperature; SE, standard error.

* *p* < .05.

**FIGURE 2:**
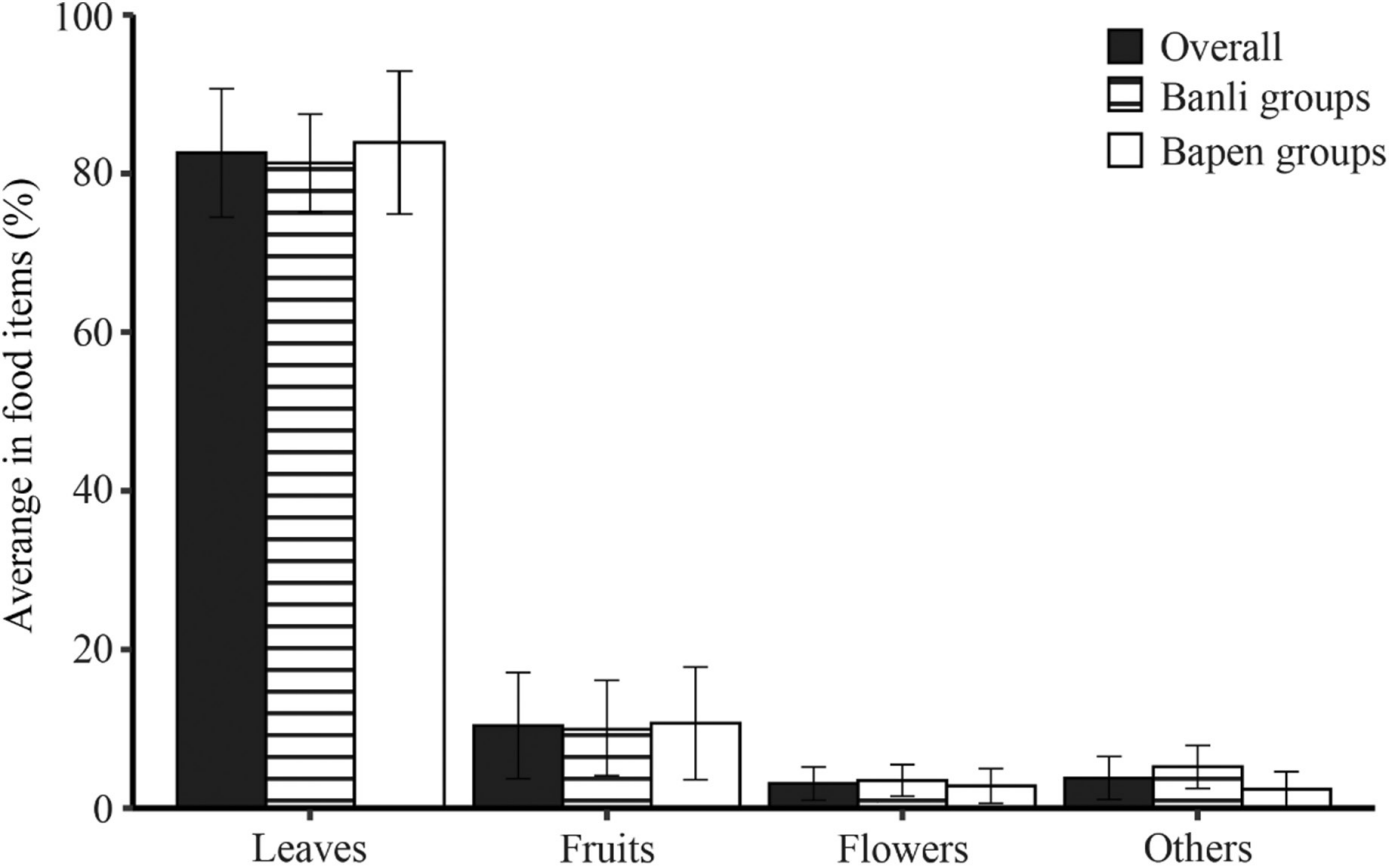
Feeding proportions of food items consumed by white‐headed langurs in limestone forests. Values are expressed as mean ± SD (significance level was set at *p* < .05, but no statistically significant difference and no significance signs were observed).

The GLMM showed no significant differences between the Banli and Bapen groups, including the feeding proportions of food items and FIDIs (leaves: *χ* ^2^ = 0.49, *df* = 1, *p* = .482; fruits: *χ* ^2^ = 0.02, *df* = 1, *p* = .876; flowers: *χ* ^2^ = 0.84, *df* = 1, *p* = .358; others: *χ* ^2^ = 3.59, *df* = 1, *p* = .058; FIDI: *χ* ^2^ = 1.35, *df* = 1, and *p* = .245). The mean feeding proportions of food items consumed by white‐headed langurs at the overall level and when grouped regionally are shown in Figure 2. On an overall level, langurs consumed leaves, fruits, flowers, and other items, which constituted 82.6%, 10.4%, 3.1%, and 3.8% of their diet, respectively. In the Banli groups, leaves accounted for 81.2% ± 6.2% of the total feeding records, followed by fruits (10.1% ± 6.0%), flowers (3.5% ± 2.0%), and other items (5.2% ± 2.7%), whereas in the Bapen groups, leaves represented 83.9% ± 9.0% of the total feeding records, followed by fruits (10.7% ± 7.1%), flowers (2.8% ± 2.2%), and other items (2.4% ± 2.2%, Figure 2).

No significant difference was found between the number of plant species consumed by langurs (*Z* = −1.04, *p* = .297) and the FDI calculated based on food species (*Z* = −0.75, *p* = .456). Additionally, the plant species most consumed by the Banli groups was kudzu (*Pueraria montana* var. *lobata*), whereas that consumed by the Bapen groups was figs (*Ficus microcarpa*) (Table 3). The kudzu was recorded and ranked in the top 10 in all studies of the Banli groups but not in those of the Bapen groups.

**TABLE 3:** Top 10 plant species consumed by the Banli and Bapen langur groups.

	Family	Species	%(*F*)
Banli groups	Leguminosae	*Pueraria montana* var. *lobata*	11.46
	Ulmaceae	*Pteroceltis tatarinowii*	6.51
	Moraceae	*Broussonetia papyrifera*	6.12
	Malvaceae	*Sterculia monosperma*	5.52
	Sapindaceae	*Boniodendron minus*	4.86
	Opiliaceae	*Cansjera rheedei*	4.18
	Convolvulaceae	*Cuscuta chinensis*	4.01
	Moraceae	*Ficus microcarpa*	3.28
	Rhamnaceae	*Ventilago inaequilateralis*	3.16
	Sapotaceae	*Sinosideroxylon pedunculatum* var. *pubifolium*	2.78
Bapen groups	Moraceae	*Ficus microcarpa*	10.30
	Ulmaceae	*Pteroceltis tatarinowii*	9.02
	Cannabaceae	*Aphananthe aspera*	5.38
	Convolvulaceae	*Cuscuta chinensis*	4.13
	Capparaceae	*Capparis membranifolia*	3.10
	Rubiaceae	*Psydrax dicocca*	2.88
	Opiliaceae	*Cansjera rheedei*	2.83
	Sapotaceae	*Sinosideroxylon pedunculatum* var. *pubifolium*	2.74
	Cannabaceae	*Celtis sinensis*	2.44
	Malvaceae	*Sterculia monosperma*	2.34

*Note*: Data of the Banli groups were extracted from Li et al. (2016), Zhang (2018), and Huang (2021). Data of the Bapen groups are cited from Li (2000), Huang (2009), Zhou et al. (2013), and Lu et al. (2021).

Abbreviation: %(*F*), Percent in feeding record.

## 4 DISCUSSION

Our findings reveal that the diets of white‐headed langurs are significantly influenced by environmental factors, including habitat fragmentation, annual rainfall, and mean annual temperature. The combination of habitat fragmentation and severe anthropogenic disturbances often leads to alterations in the microclimate of rangelands, resulting in reduced vegetation biomass and affecting the distribution patterns of animal food resources (Pyritz et al., 2010; Wilson et al., 2016). Although rainfall and temperature can shape the abundance of food resources by influencing plant growth, their impact may not prevail when habitat quality reaches a certain level of reduction (Franco et al., 2006; Mantyka‐pringle et al., 2012). Therefore, the fragmented karst mountains, coupled with frequent human modification activities, lack sufficient soil layers to support plant growth and provide essential nutrients, despite the subtropical climate offering ample rain and heat conditions for plant growth (Jiang et al., 2014; Wang et al., 2004). It is crucial to note that our findings regarding the correlation between food items consumed by langurs and environmental variables are mainly derived from the fragmentation of limestone forests. In other words, the impact of rainfall and temperature on langurs' foraging behavior is influenced by the extensive fragmentation. Therefore, investigating the potential reasons why annual rainfall and mean annual temperature affect the consumption of food items by langurs should be grounded in the context of habitat fragmentation.

Our results indicate that white‐headed langurs are affected by habitat fragmentation in feeding, with positive correlations observed between food item diversity and habitat fragmentation. Banli groups consumed more diverse food items that were experiencing higher fragmentation for the following reasons. Severe fragmentation usually indicates that the fragments become smaller, which directly impacts food availability, making dietary flexibility a critical factor for primates evolving in restricted spaces (Chaves & CÉSar Bicca‐Marques, 2013; Petersen et al., 2023). Broadening of dietary shifts is a common adaptive strategy for primates facing scarcity of food resources, involving an increased consumption of a wider range of species or food items (Bicca‐Marques, 2003; Cristóbal‐Azkarate & Arroyo‐Rodríguez, 2007). For instance, spider monkeys (*Ateles geoffroyi*) exploit a greater number of plant species in fragmented habitats than in continuous forests (Chaves et al., 2012). Black‐and‐white colobus (*Colobus guereza*) consume more ripe fruits, barks, and other items in fragments compared to unlogged forests (Chapman et al., 2007). This dietary shift may be linked to habitat fragmentation exacerbating resource scarcity within limited existence ranges. Previous studies have shown that white‐headed langurs predominantly prefer young leaves (55.7%–74.9% of total consumption records) (Li et al., 2003, 2016; Li & Elizabeth, 2006). However, their habitats were further isolated into fragments owing to natural constraints and disturbances caused by human activities (Huang et al., 2008). The difficulty of obtaining young leaves in fragmented habitats exceeds that in areas with continuous vegetation. Therefore, langurs may be compelled to consume various food items from a limited range of species, such as barks, stems, and roots. Additionally, the absence of continuous vegetation, providing shade for the fragments, exacerbates the limitations imposed by high temperatures on langur movement (Liao et al., 2021; Wessling et al., 2018). This finding might also explain the positive correlation observed between the consumption of other items and temperatures for these langurs.

Our study revealed that fruit consumption by white‐headed langurs was negatively affected by annual rainfall. Typically, a positive correlation is observed between rainfall and fruit productivity in forests (Dunham et al., 2018). Despite fruits being high‐quality food sources in karst habitats, langurs did not increase their fruit consumption in proportion to fruit availability in limestone forests (Huang et al., 2017; Zheng et al., 2021). This result might be attributed to the feeding strategy of white‐headed langurs in response to the low productivity of limestone forests. Tropical rainforests, characterized by strong productivity, ensure a more even distribution of fruits for primates, even for species primarily known as foliage eaters, which exhibit a relatively high proportion of fruit consumption (both > 30%; in this study, 10.4%), such as Banded langurs (*Presbytis femoralis*) in Malaysia and Proboscis monkeys (*Nasalis larvatus*) in Indonesia (Faudzir et al., 2021; Feilen & Marshall, 2020). Contrary to tropical rainforests, the fragmented topography of limestone forests induces a more patchy distribution of food resources, exacerbated by anthropogenic disturbances (Huang et al., 2015; Qi & Tang, 2008). In particular, canopies or vines are more likely to form connections between patches, resulting in higher availability of leaves and lower foraging costs for langurs (Bolt et al., 2021; Huang, 2002). However, langurs must expend more energy moving through patches while searching for high‐quality foods (Huang et al., 2017; Li, Ma, Zhou, & Huang, 2020). Additionally, severe anthropogenic disturbance hampers the growth of vegetation, making it difficult for them to fruit in quantity, compelling langurs to harvest evenly distributed leaves instead of high‐quality fruits (Huang et al., 2008). Further research is needed to understand the uniformity of fruit resource distribution and recovery rate in the habitat of langurs.

Notably, although habitat fragmentation significantly affects the dietary composition of white‐headed langurs, no significant difference in food items was observed between the two regions with different degrees of fragmentation. It is worth considering whether the white‐headed langur has encountered extreme conditions in both the Banli and Bapen patches, both locations have likely exceeded certain threshold of fragmentation, resulting in no significant differences (Huang et al., 2008). Despite a substantial recovery in the population size of the white‐headed langur (Tang et al., 2023), this limestone langur species remains ensnared in the dilemma of a degradated habitat.

There was no specific dataset for comparing habitat quality with primates distributed in other habitats, but the fragmentation in the limestone forests of southwestern Guangxi, China, is evident from the white‐headed langurs' pronounced reliance on leaves. In this study, white‐headed langurs derived up to 82.6% of their diet from leaves, exceeding the average value for Asian Colobines (48%) (Tsuji et al., 2013). The fragmented limestone hills, characterized by thin soils and scarce water, struggle to support various native woody plants, thereby affecting the overall abundance of fruits (Huang et al., 2017; Huang & Li, 2005). Furthermore, the fruits and flowers in subtropical regions epitomize the patchy distribution and seasonal growth of plant reproductive items (Brugiere et al., 2002; Joly et al., 2023). Therefore, in karst forests, the extreme scarcity of resources or anthropogenic disturbance and fragmentation could significantly increase langurs' dependence on foliage. This conclusion aligns with studies on sympatric François's langurs (*T. francoisi*, 52.8%–86.9%) (Hu, 2011; Li et al., 2009; Zhou et al., 2009) and Assamese macaques (*Macaca assamensis*, >70%) (Heesen et al., 2013; Huang et al., 2015; Richter et al., 2016). Similarly, rhesus macaques (*Macaca mulatta*) predominantly utilize seeds or roots in temperate forests and are frugivorous in tropical forests (Sengupta & Radhakrishna, 2016; Shao et al., 2023; Zhang et al., 2023), but they exhibit a high folivorous preference in limestone forests (Tang et al., 2011). A substantial reliance on leaves proves to be an effective strategy for these karst‐dwelling primates to adapt to limestone forests.

It is also possible that the lack of significant geographical differences in food items may result from the broad classification range, considering that the parts or vegetative and reproductive organs of plants are generally fixed. Particular attention should be given to the results related to food species. Our findings revealed no significant difference in the number of food species and FDI between the Banli and Bapen patches. However, there were significant differences in specific plant species between the two regions. For example, the Banli groups consistently consumed a substantial amount of kudzu, a behavior not observed in any studies of the Bapen groups. First, as a perennial climbing vine, kudzu is considered invasive in some studies, known for its rapid and competitive growth (Lindgren et al., 2013; Yang et al., 2014). In damaged habitats, kudzu further inhibits the development of other vegetation diversity, prolonging the habitat recovery period (Kato‐Noguchi, 2023). Second, white‐headed langurs predominantly forage in the middle and/or upper layers of vegetation (tree layers) (Huang, 2002; Liao et al., 2021). However, in areas severely affected by human disturbance, the continuity of vegetation is disrupted, increasing the proportion of aboveground lianas, represented by kudzu (Chiarello, 2003). White‐headed langurs consume large quantities of kudzu when they need to replenish young leaves through vines (Huang et al., 2017). Although the Banli groups forage more on the slopes, the lack of food supply may drive them to explore areas with relatively abundant resources, as they have been found to forage in agricultural fields (Liu et al., 2022).

The Bapen groups consistently rely on *Ficus microcarpa* as a substantial part of their diets. *Ficus* spp. has been proved to utilize calcium for growth, making it abundant in calcium‐rich limestone forests, the primary feeding ground for white‐headed langurs who predominantly consume its leaves and figs (Hu, 2011; Huang et al., 2015; O'Brien et al., 1998). However, it is consumed in a considerably smaller proportion by the Banli groups than by the Bapen groups, and in some studies within the Banli patch, it is not even the primary food source. When examining relatively stable plant resources in the habitat, if the decline in feeding is not attributable to an active change in langurs' preferences, the passive influence of other factors must be considered (Li et al., 2003). Given the land ownership constraints, reserves can only limit illegal langur hunting but cannot control local residents' land development, which includes logging or cultivation on the land surrounding the mountains in the protected area (Guangxi Forestry Bureau, 2011; Huang, 2002). Therefore, human disturbance may contribute to the loss of some trees in the Banli groups. Moreover, these two plant species indicate that the habitat of the Banli groups may still be adversely impacted by human disturbance in extreme situations. Therefore, intensified efforts are required to expedite vegetation restoration, along with the Bapen patch, which might already be experiencing severe fragmentation (Zhang et al., 2017). As white‐headed langurs contend with the gradual encroachment on their living space, ongoing sampling surveys of vegetation or sustained monitoring of dynamic changes, such as the expansion of human‐cultivated land and the reduction of natural vegetation around the active mountain, must be conducted to comprehend alterations in vegetation availability.

The present study had certain limitations. The collection and presentation of data in each study were influenced by the researcher's subjective considerations and constrained by varying research conditions. It was challenged to control variables, such as research time (rainy or dry seasons) and methods. A more thorough analysis should be conducted after acquiring ample data on food availability, thereby enhancing the accuracy of result interpretation. Despite these limitations, our study has unequivocally revealed the impact of habitat fragmentation on white‐headed langurs, offering valuable insights into the development of conservation strategies for this species and its habitat vegetation. Previous research has underscored the importance of increasing the diversity of food species as a crucial feeding strategy for limestone primates during periods or in regions where food is scarce (Zhang et al., 2017). Consequently, the conservation of white‐headed langurs should prioritize the restoration of vegetation diversity in their fragmented habitats. Additionally, native trees, which serve as natural shelter and enhance the langurs' food supply, are being felled to reclaim farmlands (Liao et al., 2021; Saldívar‐Burrola et al., 2022). Hence, protecting native vegetation and mitigating human disturbance remain critical steps in conserving langurs.

## 5 CONCLUSIONS

Habitat fragmentation significantly influences the dietary composition of white‐headed langurs. These effects must be elucidated by considering the specificity of the limestone hills, which are unable to support the growth of richer vegetation. Furthermore, both the Banli and Bapen patches may already be experiencing extreme fragmentation, as evidenced by the absence of significant differences in dietary consumptions between the langurs at these two sites. Additionally, the substantial consumption of kudzu by the Banli groups suggests ongoing frequent human disturbances, possibly through the logging of large trees, posing a potential threat to their survival. Our findings underscore the impact of habitat fragmentation on langur diets, emphasizing the fact that the development of effective conservation strategies for langurs should be premised on maintaining the continuity of the existing native vegetation and limiting further expansion of anthropogenic areas.

## AUTHOR CONTRIBUTIONS

**Ying Lai:** Formal analysis (equal); writing – original draft (lead); writing – review and editing (equal). **Yanqiong Chen:** Formal analysis (equal); writing – original draft (supporting); writing – review and editing (equal). **Kechu Zhang:** Formal analysis (equal); writing – original draft (supporting); writing – review and editing (equal). **Zhonghao Huang:** Conceptualization (lead); formal analysis (equal); methodology (lead); writing – review and editing (equal).

## CONFLICT OF INTEREST STATEMENT

None declared.

## Data Availability

All data are available in the figshare repository at https://doi.org/10.6084/m9.figshare.25367671.v1.
